# Degradation of zinc containing phosphate-based glass as a material for orthopedic tissue engineering

**DOI:** 10.1007/s10856-016-5770-x

**Published:** 2016-09-12

**Authors:** Mustafa Al Qaysi, Aviva Petrie, Rishma Shah, Jonathan C. Knowles

**Affiliations:** 1 Division of Biomaterial and Tissue Engineering, UCL Eastman Dental Institute, London, UK; 2 Department of Statistics, UCL Eastman Dental Institute, London, UK; 3 Unit of Orthodontics, Department of Craniofacial Growth and Development UCL Eastman Dental Institute, London, UK; 4 Department of Nanobiomedical Science and BK21 Plus NBM Global Research Center for Regenerative Medicine, Dankook University, Cheonan, Republic of Korea

## Abstract

Phosphate-based glasses have been examined in many studies as a potential biomaterial for bone repair because of its degradation properties, which can be controlled and allow the release of various elements to promote osteogenic tissue growth. However most of these experiments studied either tertiary or quaternary glass systems. This study investigated a qinternary system that included titanium dioxide for degradation rate control and zinc that is considered to have a role in bone formation. Zinc and titanium phosphate glass discs of different compositions were melt synthesized and samples of each composition was tested for different physical, chemical and biological characteristics via density measurement, X-ray diffraction, differential thermal analysis, mass loss, ion release, scanning electron microscopy, biocompatibility studies via live/dead assays at three time points (day 1, 4, and 7). The results showed that the glass was amorphous and that the all thermal variables decreased as zinc oxide amount raised, mass loss as well as ion release increased as zinc oxide increased, and the maximum rise was with ZnO15. The cellular studies showed that all the formulation showed similar cytocompatibility properties with MG63 except ZnO15, which displayed cytotoxic properties and this was confirmed also by the scanning electron microscope images. In conclusion, replacing calcium oxide with zinc oxide in proportion less than 10 % can have a positive effect on bone forming cells.

## 1 Introduction

Since the first synthesis of Bioglass® by L.L.Hench in 1971, various glass compositions have been developed to be examined for their suitability as biomaterials for clinical application [1]. The concept behind bioactive glass was their chemical reactivity and ability to form hydroxyl crystalline apatite on the surface of the implanted material [2]. Silicate-based glass has been used widely for hard and soft tissue repair and many studies were done to explore the use of silicate based glass for bone tissue engineering purposes and found the ability of the silicate glass scaffolds to enhance the cellular attachment and enhance both MC3T3-E1 and osteoblast cell proliferation [3, 4]. However, whilst melt derived Bioglass undergoes some initial degradation, it is relatively insoluble [5, 6], which worked as a driver to develop phosphate glass with its compositionally dependant degradation rate. In addition the components making up a phosphate-based glass may be biologically beneficial to the human body [3, 4]. The initial studies were concerned mainly with simple ternary compositions composed of phosphate, calcium oxide, and sodium oxide and that the calcium played a major role in controlling degradation, as it leads to the formation of non- bridging oxygen (P–O–Ca–O–P) bonds that lead to structural and thermal stability of the developed glass, concluding that the increase in calcium oxide percentages may reduce the degradation rate and enhance the glass chemical durability [5, 6]. However, this was followed by many studies that investigated the addition of other various elements that can play a potential role in hard tissue growth as well as enhancing glass properties such as zinc and titanium.

Zinc is not only an essential element for cell growth, proliferation, and differentiation, but it has an important role in DNA replication, growth hormones, growth factors, and enzyme production [7]. The human skeleton is usually considered the main reservoir for zinc, which is mainly stored in the osteoid layer [8, 9] It also has an influential role on bone growth and mineralization through its ability to activate aminoacyl-tRNA synthetase in osteoblastic cells, as well as preventing resorption processes due to its inhibitory action on osteoclasts [10, 11]. It was also found that zinc containing bioactive glass has the potential property to form hydroxy carbonate apatite layers on their surface after soaking in simulating body fluid [12]. Zinc phosphate-based glass was originally developed to examine its physical and biological effect on bioactive glass and was shown to enhance osteoblast like cells (HOB) cell proliferation and cellular attachment when it was added to ternary phosphate glass at different oxide mole percentages which did not exceed 5 mol % of glass composition [13]. Controversially, it displayed poor biocompatibility on MG63 when it was added to pyrophosphate glass in percentages between 10–20 % as this amount of addition may lead to increased degradation and decline in glass stability [13, 14]. Other work concerned with studying the replacement of calcium with zinc in silicate based glasses in different ratios (0.25, 0.5, 1, 1.5, 2.5, 5) mol % and revealed that zinc incorporation resulted in a lower degradation rate and enhance glass durability, which does not show significant results on cellular physiological activities [15]. The addition of zinc containing silicate glass to biphasic calcium phosphates, which is composed of hydroxyapatite and β-tri calcium phosphate showed a positive impact on rat calvarium-derived osteoblasts cell proliferation and behavior [16].

Many studies have been performed to investigate the effect of titanium addition to phosphate glass, and it was found that titanium dioxide incorporation can aid in glass stability and decrease the degradation rate and ion release [17, 18]. It was found that an optimal percentage of titanium dioxide is around 5 mol %, which has shown a biocompatible effect on MG63 cell and the addition of titanium dioxide above that amount does not have a clear and significant effect on glass degradation. The reason behind this is that titanium may tend to make resistant covalent P–O–Ti bonds instead of P–O–P that are more vulnerable to hydration [19, 20], but because of the high valency of titanium, there is a finite number of these bonds that can form.

In the current study, different compositions of quaternary and quinternary titanium stabilized zinc phosphate-based glasses were developed by the melt quenching technique , with the titanium dioxide content fixed at 5 mol % in all the compositions and zinc oxide was varied (0, 5, 10, and 15 mol %) in place of calcium oxide content to investigate the impact of adding zinc oxide on the physical and biocompatibility properties of the glass and whether the effect of titanium dioxide incorporation will decrease the degradation rate to offset the increase seen when adding zinc oxide and therefore overcome the deleterious effects of zinc incorporation that was seen in the previous study [14].

## 2 Materials and methods

### 2.1 Glass preparation

Four different glass compositions of phosphate glass were made by using phosphorus pentoxide (P_2_O_5_ 98 %, VWR, Lutterworth, UK), sodium dihydrogen phosphate (NaH_2_PO_4_, 99 %, VWR), titanium dioxide (TiO2, 99 %, VWR), calcium carbonate (CaCO_3_, 98.5 %, VWR) and zinc oxide ( ZnO, 99.95 %, Sigma-Aldrich Gillingham, UK) as precursors. The glass compositions were 50P_2_O_5_—10Na_2_O—5TiO_2_—(35-x) CaO—x ZnO (mol %) where x (zinc oxide) was 0, 5, 10, or 15mol%). The required amounts of precursor powders were weighted according to proportions by electronic balance (Sartorius) (Table 1) and blended for 1 min (Stomacher 400 blender/Seward). Once the mixing has been finished, the precursor mixture was then placed into a 200 ml volume Pt/10 %Rh crucible type 71040 (Johnson Matthey, Royston, UK) that was subsequently placed in a furnace (Carbolite) at 1350 °C for 4 h. Following this, the molten glass was then cast into a 420 °C preheated graphite mold, which was placed again in a furnace for one hour at 420 °C then left in the furnace to cool gradually to room temperature overnight to remove the residual stress, the 15 mm diameter glass rods were removed from the mould and cut into 2 mm thick glass discs using a diamond saw (Struers/ Accutom 50) and was checked it was amorphous using X-ray powder diffraction (XRD) analysis. Glass was milled to make a powder of each of the compositions for XRD and Fourier transform infrared spectroscopy analyses (MM 301 Mixer Mill, Retsch GmbH, Hope, UK).

**Table 1:** Zinc phosphate glass composition

Zinc phosphate glass composition						
Glass name and composition		Amount (g)				
		P_2_O_5_	Na_2_O	TiO_2_	CaO	ZnO
ZnO 0 %	P_50_Na_10_Ti_5_Ca_35_	56.8	24	4	35	0
ZnO 5 %	P_50_Na_10_Ti_5_Ca_30_Zn_5_	56.8	24	4	30	4
ZnO 10 %	P_50_Na_10_Ti_5_Ca_25_Zn_10_	56.8	24	4	25	8.14
ZnO 15 %	P_50_Na_10_Ti_5_Ca_20_Zn_15_	56.8	24	4	20	12.21

### 2.2 Materials characterization

#### 2.2.1 Density determination

The density of each composition was measured using an analytical balance and density determination apparatus according to Archimedes’ principal (AG 204 & MS-DNY-43, Mettler Toledo, Beaumont Leys, UK). Ethanol was used as the immersion fluid since the phosphate glasses are water-soluble. The density of the glass discs was calculated using Eq. 1:

$${\rho _{glass}} = \frac{{{M_{air}}}}{{{M_{air}} - {M_{ethanol}}}} \times {\rho _{ethanol}}$$

where *ρ*_glass_ is the density of the specimen (g/cm^3^), *ρ*_ethanol_ is the density of ethanol at the temperature at which the measurement was performed, *M*_air_ is the mass (g) of the specimen in air and *M*_ethanol_ is the mass of the specimen under ethanol.

#### 2.2.2 Thermal analysis

Differential thermal analysis was performed for the four compositions by using powder of each glass disc.

Three thermal parameters were measured: (1) *T*_*g*_ the glass transition thermal temperature, (2) *T*_*c*_ the glass crystallization temperature and (3) *T*_*m*_ the melting temperature. A Setaram differential thermal analyzer was used in this experiment using a nitrogen environment and heating temperature extended from room temperature to 1400 °C at 20 °C min^−1^.

#### 2.2.3 Material degradation

Triplicates of each composition were initially weighed then stored in plastic vials (Sterilin tube) with 25 mm pre adjusted pH deionized water to pH7 by (HCl or NH_4_OH) and incubated at 37 °C. After 1, 4, 7, and 14 days, the solution was removed and kept for ion release measurements. While the glass discs were dried and weighed at each time point to determine mass loss. Following weighing, the discs were stored again in new fresh deionized water solution.

#### 2.2.4 Ion release measurements

Ion release measurement was performed for each stored sample from the degradation study, this was done for the anions (PO_4_^3−^, P_2_O_7_^4−^, P_3_O_9_^3−^, P_3_O_10_^5−^) and the cations (Na^+^, Ca^2+^) and the transition metal (Zn^2+^) by using the ion chromatography systems (ICS1000, ICS 2500, Dionex, Thermo scientific, Hemel Hempstead, UK).

For the cation measurements all the samples were filtered prior to measurement to remove the anions (OnGuard II, Dionex).

#### 2.2.5 Cell studies

Initially, preparation of human osteoblast-like osteosarcoma cell line (MG-63, European Collection of Cell Cultures, Porton Down, UK) was done using standard conditions (37 °C, 95 % air, 5 % CO_2_, 95 % relative humidity) in Dulbecco’s modified Eagle medium (DMEM, Gibco, Life Technologies, Paisley, UK) supplemented with 10 % fetal bovine serum (Gibco) and 1 % penicillin/streptomycin (PAA Laboratories, GE Healthcare, Chalfont St. Giles, UK).

##### Cell number

**Fig. 1:**
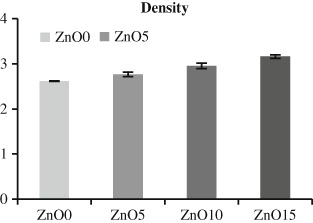
Density of phosphate-based glasses with different CaO and ZnO content. Cumulative zinc content resulted in increased glass density from 2.6 ± 0.004 g cm^−3^ for ZnO0 to 3.16 ± 0.03 g cm^−3^ for ZnO15, Error bars are SD (*n* = 3)

Three discs of each composition were sterilized by exposure to ultra-violet light for 10 min on each side before being transferred to 24 well plates on which cells were seeded evenly over the glass surface at a density of 10000 cells per disc for 15 min to allow them to properly attach to the discs then culture media was added to end up with 1 ml of culture media for each glass discs. After that all the samples were incubated at 37 °C in an atmosphere of 5 %CO_2_ for 7 days, Standard glass cover slides were used as controls. Cell culture medium was replaced at 3 day periods. Live and dead assay was performed for all the compositions by using calcien AM (4 mM) as an indicator for live cells and Ethidium homodimer-1 (2 mM) for dead cells. This was performed by initial calibration for both of the former reagents to gain the most appropriate molarity that result in the optimal fluorescent color, which was done by seeding MG63 cells on glass slides discs and preparation of various concentration of reagents as recommended by the protocol, the best results were found with 1 µM calcien and 2 µM ethidium. At each time point, culture media was removed and the cells were washed gently with Dulbecco’s phosphate buffered saline. After the washing procedure, 3 mL of calcien and ethidium mixed solution was added to each well plate and incubated for about 45 min. Following that, the solution was removed and the samples were ready for the cell counting under confocal (Bio-Rad, USA). The cell counting was done on different 13 pre identified 1 mm squares on each glass discs, these points were positioned on 15 mm circle template, which was made by transparent paper as is shown on (Fig. 1). Live and dead cells counting was performed on three times points (day 1, day 4, and day7) using the Image J software program.

##### Metabolic activity of cells

Again triplicate discs of each composition were sterilized as previously mentioned, before being transferred to 24 well plates on which cells were seeded at a density of 10,000 cells per disc in 1 ml of culture media followed by incubation at 37 °C in an atmosphere of 5 % CO_2_ for 7 days. Cell culture medium was replaced at 3 day periods and the aspirated culture was kept in another 24 cell culture plate. cytocompatibility assay was done at three times points (days 1, 4, and 7) in which a 10 % solution of water soluble tetrazolium salt-8 (alamar blue, ABD Serotec) was added to the aspirated culture then stored in the incubator for four hours at 5 % CO_2_. Alamar blue is a cell viability assay reagent which contains the cell permeable, non-toxic, and weakly fluorescent blue indicator dye called Resazurin, which is used as an oxidation-reduction (Redox) indicator that undergoes colorimetric change in response to cellular metabolic reduction. The reduced form Resorufin is pink and highly fluorescent, and the fluorescence produced is proportional to the metabolic activity of cells and was detected at 570 nm by a fluorimeter (Infinite M200, Tecan, Männedorf, Switzerland).

##### Imaging of cells

Scanning electron microscopy (SEM) images were done at the same time points for each sample which were fixed initially in 3 % glutaraldehyde followed by dehydration through a graded ethanol (50, 70, 90, 100 %) then drying by hexamethyldisilazane (Aldrich, UK).

### 2.3 Statistical analysis

Cell work data for both cell counting and metabolic activity studies were statistically assessed by hierarchal ANOVA to find the statistical significance between individual groups and the differences were considered statistically significant at *p* < 0.05.

## 3 Results

### 3.1 Density measurement

Fig. 1 shows glass density (g cm^−^3) in relation to different replacements of calcium oxides with zinc oxides and revealed that density cumulate as the content of incorporated metal oxide increase, with densities ranging from 2.6 ± 0.004 g cm^−3^ for zinc free quaternary glass to stand at 3.16 ± 0.03 g cm^−3^ for Zn15.

### 3.2 Thermal analysis

**Fig. 2:**
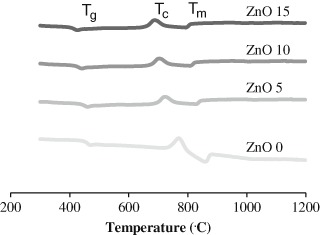
Differential thermal analysis for the whole compositions in which *T*_g_ represent glass transition temperature, *T*_c_ temperature of crystallization and *T*_m_ is melting temperature. Showing that as zinc incorporation increase all the thermal parameters decrease especially the temperature of glass transition, which decreased gradually from 471 °C for Zn0 % to 407 °C for Zn 15 %

In contrast to the density measurements, all the thermal parameters showed a decrease with increasing zinc oxide content (Fig. 2). For the glass transition temperature *T*_g_ which is considered an indicator for bulk glass properties, it ranged from about 471 °C for the ZnO 0 % to 407 °C for the Zn 15 %, likewise the crystallization temperature was at 764 °C for ZnO 0 % and decreased with increasing ZnO glass to 720 °C for 15 mol % ZnO. The same pattern was seen for the melting point in which it decreased with the incorporation of zinc oxide, it extended between the wide endothermic peak at 850 °C for the zinc oxide free glass to about 793 °C for ZnO 15 %.

### 3.3 Degradation study

**Fig. 3:**
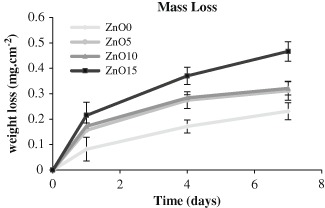
Mass loss (mg cm^−2^) for the various four zinc phosphate based glass compositions. It shows clear correlation between zinc incorporation and mass loss within time. Which was for ZnO15 about three times that of ZnO0 % in all time points. Error bars are SD (*n* = 3)

Fig. 3 shows the cumulative mass loss per unit area measured (mg cm^−2^) over a 1 week period. There was continuous mass loss over time for all the compositions but the highest rate was in the compositions with higher zinc oxide contents. In other words there was clear correlation between Zinc percentage and mass loss rate. For all the time points, the mass loss for ZnO 15 % was about three times that of Zn0 % and was about 0.46 ± 0.038 mg cm^−2^ for Zn 15 % in day 7 and about 0.16 ± 0.33 mg cm^−2^ for Zn 0 % at the same time point. Both Zn5 % and Zn 10 % had similar degradation rates at all the time points and was less than that of Zn15 %.

### 3.4 Ion release measurement

**Fig. 4:**
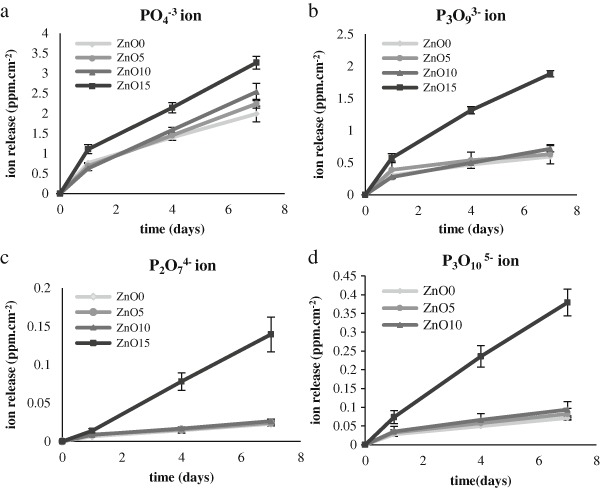
Accumulative release for anion (PPm cm^−2^) for the whole four zinc phosphate based glass compositions.**a** PO_4_^3−^, **b** P_3_O_9_^3−^, **c** P_2_O_7_^4−^, **d** P_3_O_10_^5−^. It shows clear correlation between zinc incorporation and anion within time which was obtained after incubation in deionized water for (1, 4, 7 days). Error bars are SD (*n* = 3)

**Fig. 5:**
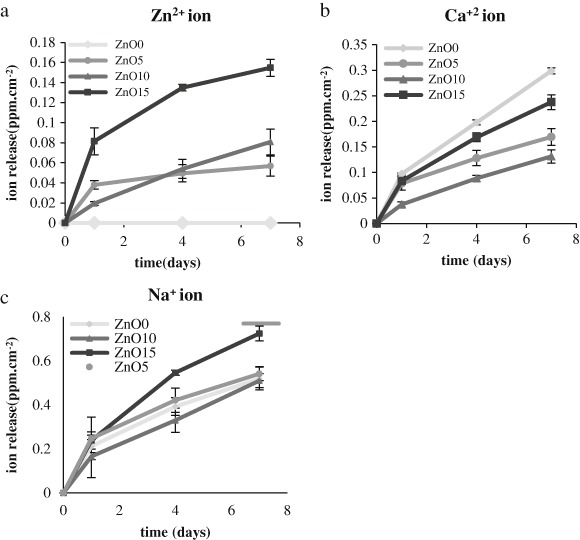
Cumulative release for anion (ppm cm^−2^) for the whole four zinc phosphate-based glass compositions. **a** Zn^2+^, **b** Ca^2+^, **c** Na^+^. It shows clear correlation between zinc incorporation and cations against time, which was obtained after incubation in deionized water for (1, 4, 7 days). Error bars are SD (*n* = 3)

Ion release results showed that anion and cation release increased with zinc composition increment as it shown in (Figs. 4, 5), respectively. Regarding the anions, ZnO 15 % displayed the highest release for all the anions, which was significantly higher than the other compositions. For the other formulations (ZnO0, ZnO5, and ZnO10), although the release was higher with the increased zinc composition, this difference was not significantly different. The cation release trend was similar to that of anions and this was clear for Zn^2+^, Ca^2+^, and Na^+^ in which cations release was significantly highest in ZnO15 and subsequently decreased as the zinc ion proportion goes down. The only exception was in Ca^2+^ release in which it was highest from the glass with the highest calcium content which is ZnO0.

### 3.5 Cytocompatibility and metabolic activity

**Fig. 6:**
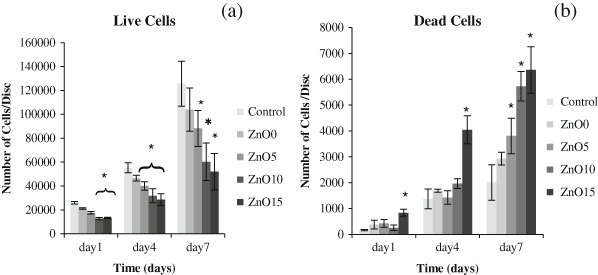
Live and dead assay by using Calcien for live cells and ethidium iodine for dead. **a** Accumulative counting for live cells displaying ZnO0 and ZnO5 has an effect that is indifferent from TCP, while there is **b** accumulative for dead. Error bars are SD (*n* = 3), While * is significant difference (*p* < 0.05)

All glass compositions showed good cytocompatibility except for ZnO15, and this was determined by the results of the cell counting assay, which involved determination of both live and dead cells from an initial seeding of around 10,000 cells/ disc. Concerning the live cells (Fig. 6a), between seeding day and day 1, cells number approximately doubled for control, ZnO0 and ZnO5 to reach about 17,500 ~ 23,000 cells/disc this was significantly more than that of ZnO10 and ZnO15, which showed a lower rise in cell density in both of them. By day 4, all the compositions displayed a further doubling to stand at about 40,000 ~ 55,000 cells/disc for control, ZnO0 and ZnO5, respectively, which was again more than that of ZnO10 and ZnO 15 that were about 30,000 cells/disc. For the last time point, cell numbers were much higher, to reach around 100,000 ~ 125,000 for the control and ZnO0 this was clearly significant for the other zinc containing glass (*p* < 0.05).

Conversely and as expected, dead cells follow the opposite pattern to that of live cells, in that dead cell numbers rise as zinc percentage increases and was maximum for ZnO15 for all the days (Fig. 6b). By day 1, dead cells number were about 160 ~ 400 cells/disc just half that of ZnO15 that was about 800 cells/disc, which was significantly higher than the other compositions. By day 4, dead cells number increased by five times for all glass types and was again significantly different in ZnO15 to the other types (*p* < 0.05). By day 7 dead cells number continued to increase especially in the zinc containing glass with maximum number of non-viable cells was in ZnO15 samples which was three times that of control samples.

**Fig. 7:**
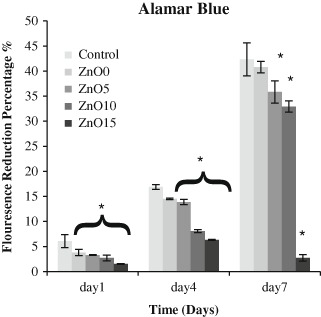
Alamar blue assay showing fluorescence reduction percentage, which is an indicator for metabolic activity. The maximum reduction was in TCP in (1, 4, 7 days), which is insignificantly different from ZnO0, ZnO5, and ZnO10. Zn15 had the lowest rate of fluorescence reduction. Error bars are SD (*n* = 3), while * is significant difference (*p* < 0.05)

Cell metabolic activity was determined by the alamar blue assay (Fig. 7) in which the reduction of fluorescence is an indicator of metabolic activity. Metabolic activity increased for all the formulations except ZnO15. At day 1, Zn0 and Zn5 showed comparable values to that for the control which was about 6 ± 1.2 % for control just slightly higher than ZnO0 and ZnO5 activity , while both ZnO10 and ZnO15 showed low reduction ability, which was significantly different from control (*p* < 0.05). By day 4, reduction rate for control increased three times to stand at 17 ± 0.4 %, which was significantly different from ZnO0, ZnO5, and ZnO10, However this statistical difference cannot neglect the existence of increase metabolic activity that happened in these glass formulations. Whereas ZnO15 show sign of significancy with the other remaining groups (*p* < 0.05).

The continuity in reduction percentage persists to day 7, which was about 42 ± 3.3 % for control and was not as high as that for ZnO0. Despite the presence of significant difference between ZnO5 and Zn10 with the control, the mean difference in reduction was little low from the control about (6.5–9 %). Interestingly, reduction rate for ZnO15 showed a clear decline in day 7 to end at 2.5 ± 0.7 % slightly higher than that at day 1 and displaying higher significant difference with the whole batches.

**Fig. 8:**
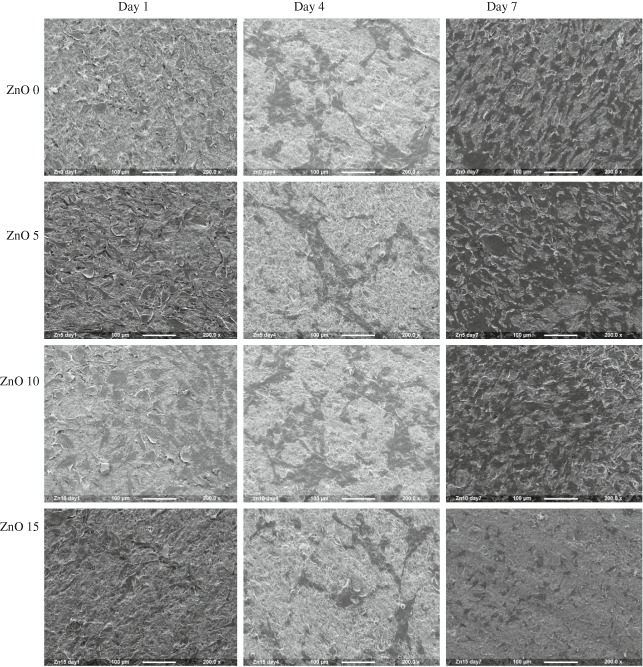
Scanning electron microscope images

SEM images (Fig. 8) showed that MG63 cells have attached to all glass compositions at all time points. After 1 day, the cells seemed to spread around the glass surface and they took an irregular shape. However, the cells on ZnO15 tended to be smaller. On day 4, the cells could be seen to have connected to each other with the appearance of rounded and swollen nucleus, some cells showed longitudinal shape that may be due to cell division. By day 7, cells were evenly distributed over the surface. Moreover, there was a clear increase in cell numbers, which were mostly interconnected with each other forming a semi-complete cellular layer. The only exception was in ZnO15 in which there was a low number of cells which were small in size and separated from each other.

## 4 Discussion

The present study concerned with the development of different phosphate-based glasses for hard tissue (bone) engineering applications, and this was done by substituting the calcium oxide with zinc oxide, since zinc is known to play important roles in the bone metabolism and development [21]. Previous studies have been carried out to assess different zinc phosphate glass properties, these studies assessed (P_2_O_5_–Na_2_O–CaO–ZnO) phosphate glass and showed that these compositions suffered from poor cytocompatibility due to their rapid degradation rate and this rate increased with increasing zinc addition [13, 14]. Other studies were performed with the incorporation of various amount of titanium dioxide to the tertiary based glass to improve its cytocompatibility and enhance its durability. titanium dioxide was added at 3 mol %, 5 mol %, 7 mol % and revealed that as titanium dioxide increased, the degradation rate and ion release rate decreased. Also the 5 mol % titanium dioxide containing glass showed the most favorable cytocompatibility results in comparison to the titanium dioxide free control samples [22] and this was the main rationale for the selection of this titanium dioxide content in the present study.

The thermal properties reduced with incorporation of zinc oxide and this tends to be as a result of the fact that zinc oxide bond enthalpy is about 284 Kj/mol, which is less than that of calcium oxide bond 464 Kj/mol as a result of this less energy is needed to break the zinc oxide bond.

Usually the main factors that control degradation of material are the atom size, bond length and electronegativity of bond. Although both of Zn–O and Ca–O bonds have pure ionic bonds [23, 24] as the difference in electronegativity of both Zn–O and Ca–O are more than 2, Ca–O seems to be more electronegative (3.44−1 = 2.44) than that of Zn-O (3.44−1.65 = 1.79) when 3.44, 1.65, and 1 are the electronegativity of O, Zn, and Ca, respectively. As a consequence, Ca–O has more bond polarity and bond strength of Zn–O, which may explain the glass susceptibility to degradation as zinc percentage increase that could affect the whole glass compositional structure and resulted in this pattern of weight loss (ZnO0 < ZnO5 < ZnO10 < ZnO15).

The ion release results were concurrent with the weight loss pattern. It was found that the increase of phosphate anion release increased as the zinc oxide content increased, this may be due to the effect of weak Zn–O–P bond. For the cation release it took the same trend for Na^+^ and Zn^+2^ ions, the only exception was for Ca^+2^ ions, which was released at higher levels in the glass with ZnO 0 mol % glass and this result was the opposite to the other ions, but that can be explained by the fact that as the ZnO content decreased, the CaO content increased. however the general information that was obtained from zinc oxide containing glass ion release was that there was a gradual release in all the ions with time which was similar for the compositions ZnO0, 5, 10 mol %, but the release for the ZnO15 mol % was higher and both findings OD mass loss and ion degradation were consistent with that of Salih et al. [13].

**Fig. 9:**
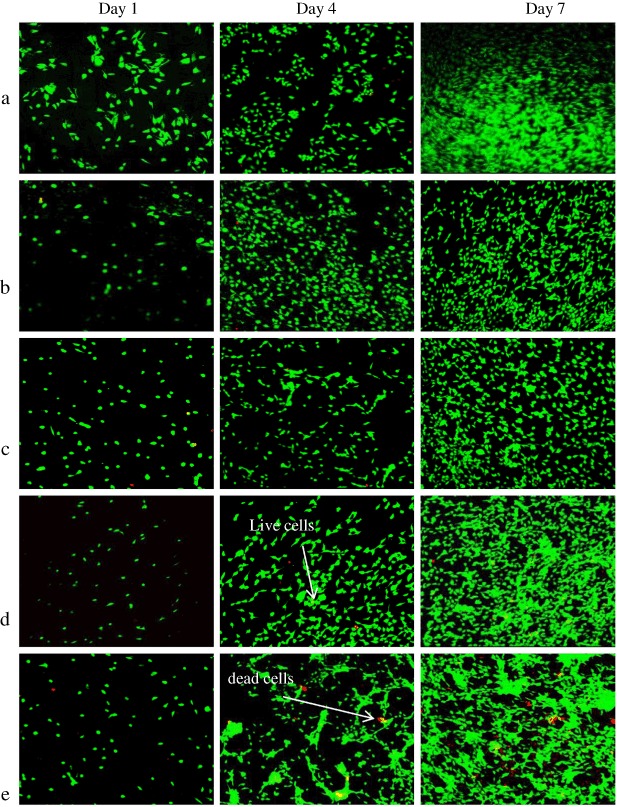
Live and dead images by using confocal. Live looks green while dead are red in color. **a** control, **b** ZnO 0, **c** ZnO5, **d** ZnO 10, **e** ZnO15

Concerning cell studies, live and dead assay was performed by using a template. Previous work was done by subjective image visualization, whereas the main aim in the current study was to objectively determine the real number of cells by using a template. The template was designed to fit the glass discs perfectly (diameter = 15 mm) and was divided into small (1 × 1 mm) squares to facilitate cell counting readings. Only 13 pre chosen squares per each sample were selected for the measurements. Although the total area of these squares constituted only 7 % of the whole disc area, their positions were selected in a way that ensure the full distribution over the whole central area of the disc to prevent repeating readings and give a valid picture of cell distribution over the disc since each of the selected squares was surrounded by non-interpreted squares. Cell seeding was performed with caution to ensure the even cell distribution for all the samples and this was by preparation of the intended cell density in 50 µl of culture media which was found to be the optimum media volume to cover the whole disc surface area. The findings of this study showed that live cell number was insignificantly different between ZnO0 and ZnO5 and the control sample for all time points, it is increased in about the same rate and even if there was slight significant difference in some time points, this does not cancel the positive impact of these two compositions on cells proliferation. Conversely, cell number was clearly low in ZnO15 and ZnO10 and was significantly different from the control group and they have about half the cell number of the control samples , this was furtherly confirmed by the results of the dead cell count which was linked to zinc oxide content (ZnO15 > ZnO10 > ZnO5 > ZnO0 > control) and this showed that the high dissolution glass gave poorer cell data, and for ZnO15 dead cells numbers constitute about 10 % of live cell numbers on the same day for the all time points which indicates cytotoxicity (Fig. 6, Fig. 9). Alamar blue measurements, is the indication of reduction–oxidation reaction (Redox) reaction as the viable metabolic cells tend to form reduced environment, which can reduce the weakly fluorescent resazurin salt to strongly fluorescent resorufin [25]. The data were concurrent with that of live and dead cells as it displayed that Zn15 have very low impacts on MG63 metabolic activity and were with highly significant difference in comparison to the other glass formulations at all time points. Despite there was little difference between ZnO10 and the control sample, it showed high metabolic activity within time. ZnO0 and ZnO5 appeared to have lower levels of effect on metabolic activity compared to the control samples and these results matched that of live cells and had the pattern of (control > ZNO0 > ZNO5 > ZnO10 > ZnO15). Despite the presence of statistical difference (*p* < 0.05) between the control and Zn0 and Zn5, there was evidence of metabolic activity enhancement in the previously mentioned glass compositions which may indicate their benign effect on cellular activity. SEM images emphasized the previous finding and showed differences in cell number for Zn15 and a decline in cellular interconnection. These outcomes may be due to the high rate of degradation in ZnO 15 %, which lead to high Zinc release which could play an unwanted impact on cell proliferation by suppression the expression mRNa as was found in previous studies [26, 27] . Interestingly, these results were very comparable with the previous study which reported that incorporation of zinc content > 1.2 % wt can lead to cytotoxic impacts on osteoblastic MC3T3-E1 cells [28], in the present study Zn composed about 1 % wt of the total glass weight in ZnO15 samples which could be due to the difference in cell type.

## 5 Conclusion

The present study showed that adding zinc to the phosphate based glass can be prepared successfully and displayed acceptable outcomes concerning cellular metabolic activities. However the increase in zinc oxide concentration to 15 mol % may have cytotoxic effects, adding zinc oxide in the percentage of 5–10 mol% may show acceptable effects when compared it with the control sample.
